# Higher physical activity levels are related to faecal microbiota diversity and composition in young adults

**DOI:** 10.5114/biolsport.2025.139850

**Published:** 2024-06-04

**Authors:** Lourdes Ortiz-Alvarez, Huiwen Xu, Samuel Ruiz-Campos, Francisco M. Acosta, Jairo H. Migueles, Ramiro Vilchez-Vargas, Alexander Link, Julio Plaza-Díaz, Angel Gil, Idoia Labayen, Jonatan R. Ruiz, Borja Martinez-Tellez

**Affiliations:** 1 PROFITH (PROmoting FITness and Health through Physical Activity) Research Group, Sport and Health University Research Institute (iMUDS), Department of Physical and Sports Education, Faculty of Sport Sciences, University of Granada, Granada, Spain; 2 Department of Biochemistry and Molecular Biology II, School of Pharmacy, University of Granada, Granada, Spain; 3 Department of Nursing, Physiotherapy and Medicine and SPORT Research Group (CTS-1024), CERNEP Research Center, University of Almería, Almería, Spain; 4 Biomedical Research Unit, Torrecárdenas University Hospital, Almería, 04009, Spain; 5 Turku PET Centre, University of Turku, Turku, Finland; 6 Turku PET Centre, Turku University Hospital, Turku, Finland; 7 InFLAMES Research Flagship Center, University of Turku, Turku, Finland; 8 Department of Biosciences and Nutrition, Karolinska Institute, Karolinska, Sweden; 9 Department of Gastroenterology, Hepatology and Infectious Diseases, Otto-von-Guericke-University Magdeburg, Magdeburg, Germany; 10 Children’s Hospital of Eastern Ontario Research Institute, Ottawa, ON K1H 8L1, Canada; 11 Institute of Nutrition and Food Technology “José Mataix”, Biomedical Research Center, Parque Tecnológico Ciencias de la Salud, University of Granada, Armilla, Granada, Spain; 12 CIBEROBN, Biomedical Research Networking Center for Physiopathology of Obesity and Nutrition, Carlos III Health Institute, Madrid, Spain; 13 Instituto de Investigación Biosanitaria, ibs.Granada, Granada, Spain; 14 Institute for Sustainability & Food Chain Innovation (ISFOOD), Department of Health Sciences, Public University of Navarra, Campus de Arrosadía, Pamplona, Spain; 15 Department of Medicine, Division of Endocrinology, and Einthoven Laboratory for Experimental Vascular Medicine, Leiden University Medical Center, Leiden, The Netherlands

**Keywords:** Physical activity, Gastrointestinal microbiome, Shorts-chain fatty acids, Obesity, Activity monitor

## Abstract

Increasing physical activity (PA) is recognised as an efficacious approach for preventing and treating cardiometabolic diseases. Recently, the composition of microorganisms living within the gut has been proposed as an important appropriate target for treating these diseases. Whether PA is related to faecal microbiota diversity and composition in humans remains to be ascertained. Thus, we examined the association of the time spent in objectively measured PA with faecal microbiota diversity and composition in young adults. A cross-sectional study enrolled 88 young adults aged 22.0 ± 2.3 years (72.7% women), whose time spent in PA at different intensities was objectively measured with a wrist-worn accelerometer for 7 consecutive days. Faecal microbiota diversity and composition were analysed with hypervariable tag sequencing of the V3–V4 region of the 16S rRNA gene. The mean Euclidean Norm of the raw accelerations Minus One (mg) during waking time, considered as overall PA, and the time spent in vigorous PA were positively correlated with alpha diversity indexes (all rho ≥ 0.23, P ≤ 0.034). Regarding faecal microbiota composition, participants with low time spent in vigorous PA had higher relative abundance of the Gammaproteobacteria class (q = 0.021, FDR = q-value) compared to the participants with high time spent in vigorous PA, and lower relative abundance of the Porphyromonadaceae family (q = 0.031) and the Alistipes genus (q = 0.015) compared to the individuals with high and intermediate time spent in vigorous PA, respectively. Our results suggest that PA, especially of vigorous intensity, is related to faecal microbiota diversity and the Gammaproteobacteria class and Porphyromonadaceae family in young adults.

## INTRODUCTION

Lifestyle physical activity (PA) is associated with a myriad of physiological adaptations that benefit human health. PA is one of the most effective strategies to prevent and combat cardiometabolic alterations and is related to a 27% decrease in mortality risk [1]. However, the underlying mechanisms that explain how PA enhances cardiometabolic health remain to be elucidated.

Gut microbiota refers to microbial communities colonising the gastrointestinal tract [2], indispensable in regulating the host nutrition, metabolic function and immunological response [3, 4]. Dysbiosis arises from an imbalance within microbial communities [5], influenced by various factors, including dietary patterns, sedentary or unhealthy lifestyles [6–8], and medication use [9]. This imbalance, in turn, correlates with conditions such as obesity and cardiometabolic diseases [10]. Recent evidence suggests that PA is one of humans’ most significant lifestyle factors influencing gut microbiota diversity and composition [11, 12]. For instance, case-controlled studies showed that faecal microbiota from athletes [13, 14] was much more diverse and had a higher proportion of several bacterial taxa than healthy sedentary individuals. Similarly, in football athletes, it was found that increased levels of PA promoted greater diversity of the faecal microbiota via the production of short-chain fatty acids by gut bacteria, enhancing overall health [15]. Another cross-sectional study observed that premenopausal women meeting the PA World Health Organization recommendations had a greater relative abundance of *Akkermansia* and *Faecalibacterium* genera than sedentary women [16]. Evidence indicates that *Akkermansia* and *Faecalibacterium* genera are associated with reduced inflammation and, therefore, may play a role in preventing the development of cardiometabolic diseases [17]. Based on that, a recent study showed that individuals with higher levels of PA showed a different Mediterranean pattern and faecal microbiota composition than individuals with obesity who reported lower levels of PA [18]. Most studies investigating the relationship between PA and faecal microbiota composition have used self-reported questionnaires to determine PA levels [13, 14, 19]. However, these instruments have the disadvantage of misclassifying PA levels and thus compromise the ability to detect valid associations between PA levels and faecal microbiota composition [20]. Based on the aforementioned studies using self-reported data, we hypothesise that increased levels of PA, at different intensities, are associated with elevated faecal microbiota diversity and a greater prevalence of beneficial bacteria. Thus, through the utilization of objective measures of PA in the present study, we aimed to explore the association between the time spent in objectively measured PA at different intensities with faecal microbiota diversity and composition in a cohort of young individuals.

## MATERIALS AND METHODS

### Design study and participants

A total of 92 (65 women) young healthy adults, aged 18–25 years, were included in the present cross-sectional study. This study was carried out within the framework of the ACTIBATE study [21], an exercise-based randomized controlled trial (Clinical Trials.gov ID: NCT02365129). All assessments were performed in Granada (Spain) between October and November in 2016. Inclusion criteria were: being engaged in less than 20 min of moderate-vigorous PA on less than 3 days/week, having a stable body weight over the last 3 months (< 3 kg change), not smoking, not taking any medication (including antibiotics in the last 3 months), not presenting any acute or chronic illness and not being pregnant. The study protocol and experimental design were applied in accordance with the last revised ethical guidelines of the Declaration of Helsinki. The study was approved by the Ethics Committee on Human Research of the University of Granada (no. 924) and the Servicio Andaluz de Salud (Centro de Granada, CEI-Granada); all participants signed informed consent.

### Physical activity assessment

PA variables were objectively measured with one accelerometer on the non-dominant wrist (ActiGraph GT3X+, Pensacola, FL), during 7 consecutive days (24 h/day) [21]. Detailed information about how to wear the accelerometer was given to participants, including the instruction to remove it in daily water-based activities, such as washing dishes or showering.

The sampling frequency of 100 Hz was selected to store the raw accelerations of the accelerometers [22]. We exported and converted the raw accelerations to the “.csv” format using ActiLife v.6.13.3 software (ActiGraph, Pensacola, FL, US). Afterwards, the “ggir” [23] package in R software was used to process the raw “.csv” files. This processing consisted of: (i) local gravity data auto-calibration of accelerations according to the local gravitational acceleration [24], (ii) calculation of the Euclidean Norm of the raw accelerations Minus One *G* with negative values rounded to 0 (ENMO) calculated elsewhere [25], (iii) detection of non-wear time based on the raw acceleration of the three axes, (iv) determination of MAL detection of sustained functioning of the accelerometer by means of abnormal high accelerations incompatible with human movement (i.e., related to device malfunctioning), (v) imputation of non-wear time and abnormal high accelerations, (vi) identification of waking and sleeping time based on the automatized algorithm guided by the participants’ daily reports [26], and (vii) estimation of sedentary time and the time spent in light PA, moderate PA, vigorous PA, and moderate to vigorous PA using agespecific cut-points for a wrist-worn accelerometer, for Euclidean Norm Minus One (ENMO) [27]. We measured the mean ENMO (m*g*) during waking time, which is considered an overall indicator of the PA (overall PA). For the analyses we only included the participants who wore the accelerometers for ≥ 16 h/day during at least 4 days (including at least 1 weekend day).

### Faecal microbiota analyses

#### Stool collection and DNA extraction

The participants collected approximately 50 g of a faecal sample in plastic sterile containers, which were transported in portable coolers to the research centre. Faecal samples were stored at -80°C until extraction of DNA. The QIAamp DNA Stool Mini Kit (QIAGEN, Barcelona, Spain) was used for extraction of DNA, following the manufacturer’s instructions. The samples were incubated at 95ºC to ensure lysis of both gram-positive and gram-negative bacteria. Then, we quantified DNA with a NanoDrop ND1000 spectrophotometer (Thermo Fisher Scientific, DE, USA). Finally, DNA purity was determined by measuring the ratio of absorbance at A260/280 nm and A260/230 nm.

### Sequencing analysis

DNA extracted was amplified by polymerase chain reaction (PCR) by primer pairs – forward primer (5’CCTACGGGNGGCWGCAG3’) and reverse primer (5’GACTACHVGGGTATCTAATCC3’) – targeting the V3 and V4 hypervariable regions of the bacterial 16S rRNA gene. All PCRs were executed in 25 µL reaction volumes incorporating 12.5 µL of 2X KAPA HiFi Hotstart ready mix (KAPA Biosystems, Woburn, MA, USA), 5 µL of each forward and reverse primer (1 µM) and 2.5 µL of extracted DNA (10 ng) under the following cycling circumstances: (a) denaturation at 95°C for 3 min, (b) cycles of denaturation at 95°C for 30 s, (c) annealing at 55ºC for 30 s, (d) elongation at 72ºC for 30 s , (e) a final extension at 72°C for 5 min. To purify the PCR products from free primers and primer dimers we used AMPure XP beads (Beckman Coulter, Indianapolis, IN, USA). Next, the index PCR attached dual indices and Illumina sequencing adapters using the Nextera XT Index Kit (Illumina, San Diego, CA, USA), on a thermal cycler using the requirements previously mentioned. After that, AMPure XP beads (Beckman Coulter, Indianapolis, IN, USA) were used for purification of the pooled PCR products. The resultant amplicons were sequenced at MiSeq (Illumina, USA), using a paired-end (2 × 300 nt) Illumina MiSeq sequencing system (Illumina, San Diego, CA, USA).

### Bioinformatics analysis

We analysed the FASTQ files with the “dada2” [28] package in R software, obtaining 11,659,014 paired ends with an average of 126,728 ± 33,395 reads per sample. The cut-off of 10,000 reads was surpassed for all samples. Samples were resampled to a minimum sequencing depth of 30,982 reads using the “phyloseq” [29] package in R software, returning 11,158 phylotypes.

The “Classifier” function from the Ribosomal Database Project (RDP) was used to assign the taxonomic affiliation of phylotypes, based on the naïve Bayesian classification [30] with a pseudo-bootstrap threshold of 80%. We obtained a total of 209 genera belonging to 16 different phyla. The “seqmatch” [31] function from RDP was performed to define the discriminatory power of each sequence read with the purpose of annotating species assignments; we executed annotation according to previously published criteria [32]. Microbial communities were analysed at different taxonomic levels (phylum to genus), calculating relative abundances, expressed as percentages.

We performed the analyses with those bacteria with more than 0.5% on average in their relative abundance.

Next, alpha and beta diversities were estimated based on the identified microbial communities. Alpha diversity takes into account the number of different phylotypes and relative abundances of a single sample [33], whereas beta diversity shows differences in microbial community composition between individuals, which is the degree to which samples differ from one another [34]. Alpha diversity was assessed based on Chao richness, Shannon, inverse Simpson and evenness Camargo indexes with the “microbiome” [35] package in R software. Chao richness estimates diversity according to the number of different phylotypes in the community [36]; that is, higher Chao richness indicates higher diversity in the community. Shannon diversity increases as both the richness and the evenness of the community increase [37]; the inverse of Simpson diversity is calculated from the classical Simpson diversity and indicates richness in a community with uniform evenness [38], and evenness Camargo determines the equitability of phylotype frequencies in a community [39]. Beta diversity was measured quantitatively using permutational multivariate analysis of variance (PERMANOVA) based on Bray-Curtis dissimilarity.

### Anthropometric and body composition measurements

Participants’ weight and height were measured, without shoes and wearing the standard clothes, using a SECA scale and stadiometer (model 799, Electronic Column Scale, Hamburg, Germany). Body mass index (BMI) was calculated as weight (kg)/height (m^2^). Body composition was evaluated by dual energy X-ray absorptiometry (DEXA, HOLOGIC, Discovery Wi, Marlborough, MA). The lean mass index (LMI) and fat mass index (FMI) were calculated as lean body mass and fat body mass, respectively, in kg, divided by height in m^2^. The fat mass percentage was determined as the body fat mass divided by the total body mass and multiplied by 100.

### Cardiometabolic profile

Fasting blood samples were collected for assessment of the cardio-metabolic profile. Serum glucose, total cholesterol, high density lipoprotein-cholesterol (HDL-C) and triglycerides were measured following standard methods using an AU5832 automated analyser (Beckman Coulter Inc., Brea CA, USA). Low-density lipoprotein cholesterol (LDL-C) was estimated as: *[total cholesterol – HDL-C – (triglycerides/5)]*, in mg/dL. Serum insulin was measured using the Access Ultrasensitive Insulin chemiluminescent immunoassay kit (Beckman Coulter Inc., Brea CA, USA). The homeostatic model assessment for insulin resistance (HOMA-IR) index was calculated as *(insulin (µU/mL) × glucose (mmol/L)/22.5*.

### Dietary assessment

Dietary intake was registered using three non-consecutive 24-hour recalls, 2 weekdays and a weekend day. These 24-hour recalls were performed in the laboratory via face-to-face interviews with dietitians. To improve the accuracy of food quantification, we used coloured photographs of different portion sizes of food during the interviews [40]. All 24-hour recalls were analysed for total energy (kcal), fat, proteins, carbohydrates, and fibre intake (g) by EvalFINUT software, which is based on the United States Department of Agriculture (USDA) and “Base de Datos Española de Composición de Alimentos” (BEDCA) databases.

### Statistical analysis

This is a secondary study derived from the ACTIBATE trial [41]; therefore, there is not a sample size calculation for this study. Data normality was explored using the D’Agostino & Pearson omnibus, visual histograms and Q-Q plots (data not shown). None of the variables followed a normal distribution; therefore data were presented as median ± interquartile range and non-parametric tests were used for all analyses. Moreover, no sex interaction was detected (all P > 0.05), so both sexes were pooled together. Spearman correlations were performed to investigate the correlation between the PA variables and faecal microbiota diversity, using the “psych” [42] and “corrplot” [43] packages in R software. Since faecal microbiota diversity can be modified by several factors including sex [44], BMI [45] and dietary intake [46], we repeated the aforementioned correlations adjusted for sex, BMI and dietary intake in separate models (data not shown). Moreover, we repeated this analysis by adjusting for accelerometer non-wear time and glucose levels in separate models (data not shown) as possible confounders of PA variables. Overall PA and the time spent in vigorous PA were computed as tertiles according to number of participants with SPSS (SPSS v. 22.0, IBM SPSS Statistics, IBM Corp. Armonk, NY), because they were the only variables with a significant correlation with faecal microbiota diversity. The tertiles’ values for overall PA were low (13.45–29.44 m*g*), intermediate (30.02–35.41 m*g*), and high (35.49–67.10 m*g*), whereas for the time spent in vigorous PA the values were low (0.02–0.83 min/day), intermediate (0.87–2.67 min/day), and high (2.75–14.40 min/day). Tertiles of overall PA and the time spent in vigorous PA were compared using one-way PERMANOVA with 9,999 permutations for significance testing with the Paleontological Statistics (Past3) software [47] for the calculation of beta diversity. Kruskal-Wallis tests were performed to investigate whether there were significant differences in body composition, dietary intake and cardiometabolic profile as well as faecal microbiota alpha diversity and composition outcomes across tertiles of overall PA and the time spent in vigorous PA. Analysis of covariance was used to compare the relative abundance of genera across tertiles of the time spent in vigorous PA adjusted for protein intake with the data transformed by Blom’s formula. All P values were corrected by Benjamini and Hochberg multiple testing to control the false discovery rate (FDR, shown as q-values) [48]. The level of significance was set at P < 0.05 and q < 0.05. R software (V.3.6.0; http://www.r-project.org) and GraphPad Prism version 8.0.0 for Windows (GraphPad Software, San Diego, California, USA, (http://www.graphpad.com) were used for the statistical analysis and graphical plots.

## RESULTS

### Characteristics of participants

A total of 92 participants had data from analysis of faecal microbiota diversity and composition, but only 88 participants (24 men, age = 22.0 ± 2.0; and 64 women, age = 21.6 ± 2.0) had valid PA measurements (as they wore the accelerometer for < 16 h/day during at least 4 days), who were finally included in the analyses. Table 1 shows the descriptive characteristics of the included participants (age 21.7 (19.8–23.9) years and BMI 23.6 (21.6–28.1 kg/m^2^)), of whom 72.7% were women. We performed tertiles of overall PA and the time spent in vigorous PA and we observed that, generally, body composition, dietary intake and cardiometabolic profile were similar across them (Table S1), with the exception of protein intake and glucose levels (P = 0.018 and P = 0.003, respectively; Table S1).

**TABLE 1:** Descriptive characteristics of the participants.

	All (N = 88)	Men (N = 24)	Women (N = 64)
Age (years)	21.7 (19.8–23.9)	22.0 (19.8–24.5)	21.6 (19.8–23.7)

*Body composition*			
Body mass index (kg/m^2^)	23.6 (21.6–28.1)	25.8 (22.8–32.5)	23.3 (21.0–27.4)
Lean mass index (kg/m^2^)	13.9 (12.6–15.7)	17.4 (15.3–18.4)	13.2 (12.5–14.3)
Fat mass index (kg/m^2^)	8.7 (6.3–11.5)	7.9 (4.5–11.3)	9.2 (6.5–11.5)
Fat mass (%)	36.7 (31.2–42.6)	32.5 (20.8–37.1)	39.3 (32.9–42.8)

*Sedentary and physical activity time*			
Non wear time (min/week)	8.6 (0.0–22.2)	8.3 (0.0–28.9)	8.6 (0.0–22.2)
Overall PA (m*g*)	31.8 (27.2–37.2)	31.0 (24.8–35.3)	32.8 (28.2–37.4)
Sedentary Time (min/day)	785.2 (753.2–820.7)	798.8 (770.0–837.7)	779.2 (752.6–820.2)
Light PA (min/day)	113.6 (99.2–139.0)	109.8 (90.7–124.2)	118.8 (99.7–145.3)
Moderate PA (min/day)	90.0 (67.9–109.5)	84.1 (57.7–103.1)	93.1 (69.7–113.1)
Vigorous PA (min/day)	1.2 (0.7–3.8)	1.3 (0.5–3.3)	1.2 (0.7–4.6)
Moderate to Vigorous PA (min/day)	91.3 (70.5–111.3)	86.3 (58.1–104.6)	95.6 (72.2–116.4)

*Dietary intake*			
Energy intake (kcal/day)	1847.7 (1580.8–2184.4)	2085.2 (1783.3–2641.3)	1798.2 (1553.6–2090.2)
Fat (g/day)	84.5 (70.0–100.2)	97.8 (71.5–111.8)	82.1 (69.2–96.7)
Proteins (g/day)	72.6 (61.4–90.9)	102.2 (75.3–118.9)	67.0 (59.8–79.4)
Carbohydrates (g/day)	198.2 (159.3–229.6)	200.4 (172.5–244.9)	198.2 (149.2–226.2)
Fiber (g/day)	16.2 (11.7–19.8)	16.3 (13.4–20.5)	16.2 (11.2–19.4)

*Cardiometabolic profile*			
Glucose (mg/dL)	87.0 (84.0–92.0)	91.0 (85.0–97.0)	87.0 (84.0–92.0)
Insulin (µUI/mL)	6.7 (4.9–10.7)	6.9 (4.8–12.4)	6.7 (4.9–9.5)
HOMA index	1.4 (1.1–2.4)	1.5 (1.0–3.0)	1.4 (1.1–2.2)
Total Cholesterol (mg/dL)	161.0 (141.5–189.0)	153.0 (139.0–174.0)	166.0 (143.0–197.0)
HDL-C (mg/dL)	51.0 (46.0–58.3)	46.0 (40.0–51.0)	53.0 (48.0–63.0)
LDL-C (mg/dL)	94.0 (78.0–114.0)	90.0 (81.0–104.0)	96.0 (78.0–114.0)
Triglycerides (mg/dL)	71.0 (52.8–106.3)	77.0 (60.0–115.0)	68.0 (52.0–98.0)

*Faecal microbiota*			
***Alpha diversity indexes***			
Richness Chao	391.6 (339.8–508.1)	374.1 (333.9–448.1)	423.5 (344.1–538.7)
Shannon diversity	4.2 (4.0–4.5)	4.2 (4.0–4.5)	4.3 (4.0–4.5)
Inverse Simpson diversity	31.7 (23.1–42.7)	31.7 (22.5–49.1)	31.7 (23.2–41.5)
Evenness Camargo	0.2 (0.2–0.3)	0.2 (0.2–0.3)	0.2 (0.2–0.3)

***Composition (Phylum)***			
*Actinobacteria* (%)	1.1 (0.6–1.9)	1.1 (0.4–1.7)	1.1 (0.6–2.2)
*Bacteroidetes* (%)	41.3 (34.7–44.8)	41.8 (39.9–45.2)	40.9 (34.3–44.8)
*Firmicutes* (%)	47.2 (42.1–52.5)	48.7 (43.3–52.0)	45.6 (41.3–53.1)
*Proteobacteria* (%)	5.1 (3.8–8.2)	6.7 (4.2–8.8)	4.6 (3.5–7.3)
*Verrucomicrobia* (%)	0.1 (0.0–2.0)	0.0 (0.0–0.8)	0.2 (0.0–3.5)

Data are presented as median (interquartile range). BMI: body mass index; FMI: fat mass index; HDL-C: high-density lipoprotein cholesterol; HOMA index: homeostatic model assessment index; LDL-C: low-density lipoprotein cholesterol; LMI: Lean mass index; m*g*: mili-gravitational units; PA: Physical activity.

### Relationship between physical activity and faecal microbiota diversity

**FIG. 1:**
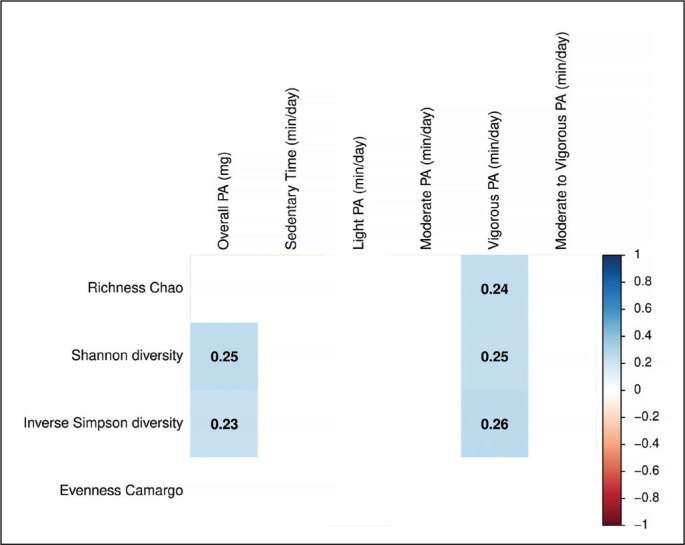
Spearman correlation of physical activity variables with faecal microbiota diversity. Boxes only represent the statistically significant (P < 0.05) correlations and the values within the boxes show the Spearman correlation coefficient. Blue boxes indicate a positive correlation whereas red squares indicate a negative correlation between physical activity variables and faecal microbiota diversity indexes. m*g*: milli-gravitational units; PA: physical activity.

Overall PA and the time spent in vigorous PA were positively correlated with alpha diversity indexes, more specifically with Shannon and inverse Simpson diversity indexes (all rho ≥ 0.23, P ≤ 0.034; Fig. 1). Only the time spent in vigorous PA was positively correlated with the Chao richness index (rho = 0.24, P = 0.023; Fig. 1). However, we did not observe any significant correlation between other PA variables and alpha diversity indexes (all P > 0.05; Fig. 1). The results were similar when sex, BMI, energy and macronutrient intake, as well as accelerometer non-wear time and glucose levels, were included as confounders in separate models (data not shown). Moreover, we found that individuals with high time spent in vigorous PA had a higher Chao richness, Shannon and inverse Simpson diversity indexes than individuals with low and intermediate time spent in vigorous PA (all P ≤ 0.038; data not shown). However, there were no differences across tertiles of overall PA and the time spent in vigorous PA in the beta diversity at any taxonomic levels (all P ≥ 0.060; Table 2).

**TABLE 2:** Beta diversity across tertiles of overall PA and the time spent in vigorous PA at all taxonomic levels.

	Tertiles of overall PA (mg)		Tertiles of the time spent in vigorous PA (min/day)	

*Taxonomic levels*	*Pseudo-F*	*P*	*Pseudo-F*	*P*
Phylum	0.502	0.789	0.967	0.443
Class	0.926	0.508	1.224	0.262
Order	0.959	0.475	1.282	0.221
Family	1.132	0.306	1.476	0.106
Genus	0.968	0.492	1.524	0.060

PERMANOVA using 9,999 permutations for significance testing (p-value < 0.05). m*g*: mili-gravitational units; PA: physical activity; Pseudo-F: statistic, larger number indicate greater separation [64] across tertiles of total PA and vigorous PA levels.

### Relationship between physical activity variables and faecal microbiota composition

**FIG. 2:**
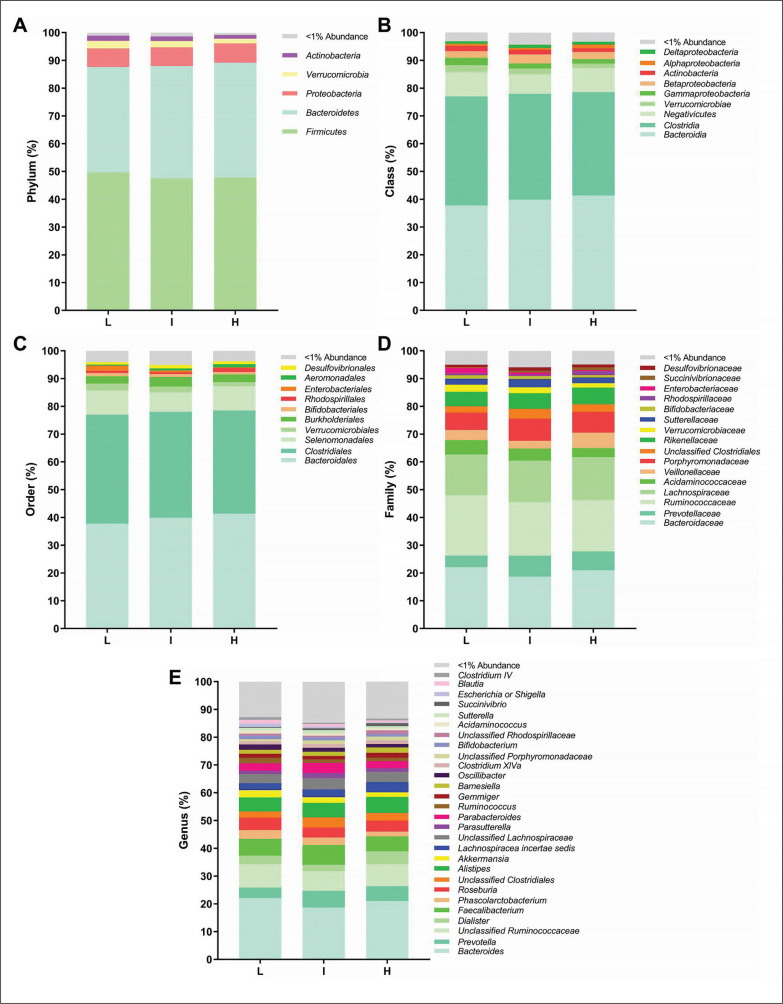
Faecal microbiota composition according to tertiles of overall physical activity (PA) levels (L: low, 13.45–29.44 m*g*; I: intermediate, 30.02–35.41 m*g*; H: high, 35.49–67.10 m*g*). Panels indicate relative abundance of the faecal microbiota at phylum (A), class (B), order (C), family (D) and genus (E) taxonomic levels according to tertiles of overall PA. Stacked bar represents percentage abundance. Kruskal-Wallis test was used to test for each pairwise comparison, correcting for multiple comparisons FDR (q < 0.05) (GraphPad Prism 8.00).

**FIG. 3:**
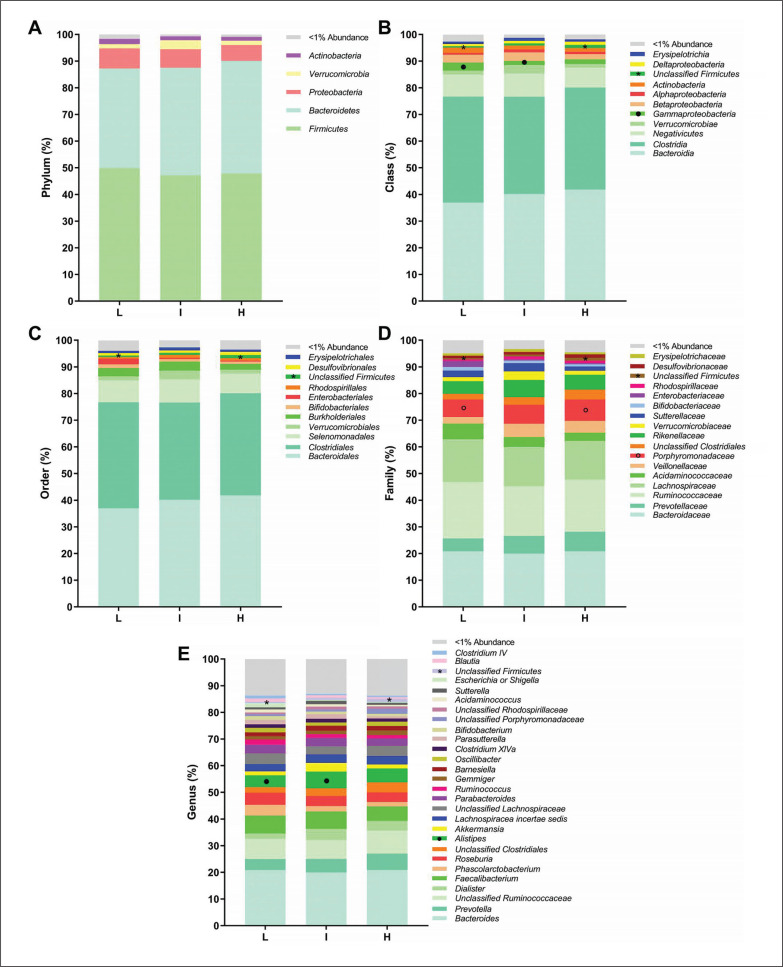
Faecal microbiota composition according to tertiles of the time spent in vigorous physical activity (PA) (L: low, 0.02–0.83 min/day; I: intermediate, 0.87–2.67 min/day; H: high, 2.75–14.40 min/day). Panels indicate relative abundance of the faecal microbiota at phylum (A), class (B), order (C), family (D) and genus (E) taxonomic levels according to tertiles of the time spent in vigorous PA. Stacked bar represents percentage abundance. Symbols * and ◦ mean statistical significance differences between low and high time spent in vigorous PA, and symbol • represents statistical significance differences between low and intermediate time spent in vigorous PA. Kruskal-Wallis test was used to test for each pairwise comparison, correcting for multiple comparisons FDR (q < 0.05) (GraphPad Prism 8.00).

We analysed the differences across tertiles of overall PA and the time spent in vigorous PA on faecal microbiota composition at all taxonomic levels. There were no significant differences across tertiles of overall PA on the relative abundance of all bacteria at the different taxonomic levels (all P > 0.05; Fig. 2). Similarly, we observed no differences across tertiles of time spent in vigorous PA on the relative abundance of bacteria at the phylum taxonomic level (all P ≥ 0.318; Fig. 3A). However, we observed that individuals with low time spent in vigorous PA had a higher relative abundance of the *Gammaproteobacteria* class (*Proteobacteria* phylum) than individuals with intermediate time spent in vigorous PA (q = 0.021, FDR = q-value; Fig. 3B). Moreover, individuals with high time spent in vigorous PA had higher relative abundance of unclassified *Firmicutes* class (*Firmicutes* phylum) and *Porphyromonadaceae* family (*Bacteroidetes* phylum) than individuals with low time spent in vigorous PA (q = 0.027, and q = 0.031, respectively; Fig. 3B and D). Finally, we found that individuals with intermediate time spent in vigorous PA had a higher relative abundance of the *Alistipes* genus (*Bacteroidetes* phylum) than individuals with low time spent in vigorous PA (q = 0.015; Fig. 3E). Interestingly, these same participants presented differences in protein intake (q = 0.011; Table S1). Thus, we repeated the analyses after adjusting for the protein intake and the differences in the relative abundance of the *Alistipes* genus disappeared (P = 0.080; Table S2).

**TABLE S1:** Characteristics of participants according to tertiles of overall PA and the time spent in vigorous PA.

	Overall PA (mg)				Time spent in vigorous PA (min/day)			

	Low (13.5–29.4) n = 29	Intermediate (30.0–35.4) n = 30	High (35.5–67.1) n = 29	P	Low (0.0–0.8) n = 29	Intermediate (0.9–2.7) n = 30	High (2.8–14.4) n = 29	P
Age (years)	22.3 ± 2.5	22.0 ± 2.0	21.6 ± 2.5	0.461	22.3 ± 2.3	22.2 ± 2.3	21.4 ± 2.2	0.239
Sex, N				0.501				0.555
Men	10	8	6		8	10	6	
Women	19	22	23		21	20	23	

*Body composition*								
BMI (kg/m^2^)	25.6 ± 5.4	24.2 ± 4.7	25.2 ± 4.2	0.548	24.5 ± 5.0	25.7 ± 4.8	24.8 ± 4.6	0.531
LMI (kg/m^2^)	14.4 ± 2.5	13.9 ± 2.3	14.9 ± 2.0	0.150	14.0 ± 2.3	14.5 ± 2.2	14.6 ± 2.4	0.380
FMI (kg/m^2^)	9.3 ± 3.4	8.7 ± 2.7	8.9 ± 3.3	0.805	9.0 ± 3.5	9.1 ± 3.0	8.8 ± 2.9	0.946
Fat mass (%)	37.1 ± 7.7	36.7 ± 6.5	35.4 ± 8.9	0.823	36.8 ± 8.5	36.6 ± 7.5	35.7 ± 7.3	0.734

*Dietary intake*								
Energy intake (kcal/day)	1985 ± 597.1	1805 ± 382.1	1992 ± 453.2	0.294	1827 ± 602.5	2055 ± 412.1	1892 ± 411.0	0.080
Fat (g/day)	91.0 ± 36.7	81.4 ± 21.9	89.3 ± 22.8	0.344	82.6 ± 37.2	92.1 ± 23.8	86.6 ± 20.0	0.171
Proteins (g/day)	78.9 ± 27.2	75.2 ± 19.6	80.6 ± 23.0	0.597	71.3 ± 24.6*	85.2 ± 22.4*	77.9 ± 21.3	**0.018**
Carbohydrates (g/day)	208.6 ± 61.5	188.1 ± 57.5	211.7 ± 67.3	0.215	196.7 ± 67.6	216.0 ± 51.5	194.8 ± 67.0	0.129
Fiber (g/day)	16.9 ± 6.2	16.8 ± 8.0	17.0 ± 5.1	0.529	16.6 ± 7.2	17.7 ± 6.7	16.4 ± 5.6	0.654

*Cardiometabolic profile*								
Glucose (mg/dL)	89.2 ± 7.5	88.7 ± 6.1	86.8 ± 6.4	0.502	89.6 ± 6.3*	**90.3 ± 7.0†**	84.7 ± 5.5*†	**0.003**
Insulin (µUI/mL)	10.8 ± 9.3	7.4 ± 3.3	7.5 ± 4.3	0.149	9.4 ± 6.2	9.3 ± 8.1	7.0 ± 3.9	0.139
HOMA index	2.5 ± 2.6	1.6 ± 0.8	1.6 ± 1.0	0.132	2.1 ± 1.6	2.2 ± 2.3	1.5 ± 0.9	0.059
Total Cholesterol (mg/dL)	171.8 ± 38.1	167.6 ± 32.6	167.0 ± 42.1	0.757	163.0 ± 32.9	179.9 ± 47.2	163.3 ± 27.4	0.370
HDL-C (mg/dL)	51.4 ± 14.1	54.4 ± 9.3	52.6 ± 12.1	0.241	51.0 ± 13.0	55.2 ± 12.6	52.3 ± 9.8	0.448
LDL-C (mg/dL)	100.8 ± 29.1	97.5 ± 27.0	98.7 ± 29.1	0.893	95.0 ± 25.6	105.4 ± 34.1	96.5 ± 23.2	0.599
Triglycerides (mg/dL)	100.6 ± 65.2	89.6 ± 63.4	80.7 ± 63.0	0.068	84.6 ± 46.9	113.8 ± 91.9	72.3 ± 27.5	0.242

Data are presented as means ± standard deviations. Symbol (*) indicates significant differences between low and high tertiles, whereas symbol (†) shows significant differences between intermediate and high tertiles by means of Kruskal-Wallis. BMI: body mass index; FMI: fat mass index; HDL-C: high-density lipoprotein cholesterol; HOMA index: homeostatic model assessment index; LDL-C: low-density lipoprotein cholesterol; LMI: Lean mass index; m*g*: mili-gravitational units; PA: Physical activity.

**TABLE S2:** Differences in the relative abundance of *Alistipes* genus across tertiles of the time spent in vigorous PA.

Time spent in vigorous PA (min/day)					

	Low (0.0–0.8) n = 29	Intermediate (0.9–2.7) n = 30	High (2.8–14.4) n = 29	P for model 1	P for model 2
*Alistipes* genus (%)	4.3 ± 2.9*	6.3 ± 3.3*	5.2 ± 3.0	**0.026**	0.080

Data are presented as means ± standard deviations. Symbol (*) indicates significant differences between low and intermediate tertiles. Model 1: P value from Kruskal Wallis test. Model 2: P values were obtained from one-way analyses of variance adjusted for protein intake (g) with data transformed by Blom’s formula. PA: Physical activity.

### DISCUSSION

In the present study, overall PA and the time spent in vigorous PA were found to be positively correlated with alpha diversity indexes in young adults. Moreover, there were differences across the tertiles of time spent in vigorous PA in the relative abundance of the *Gammaproteobacteria* class (*Proteobacteria* phylum), *Porphyromonadaceae*family and *Alistipes* genus (both *Bacteroidetes* phylum). These findings indicate that PA may play a role in faecal microbiota diversity and composition in young adults, although further studies are needed to confirm these findings.

Our results showing the positive correlation between PA and alpha diversity agree with recent findings [14, 49, 50]. However, the mechanisms by which PA may promote higher faecal microbiota diversity are unknown. A possible explanation could be the changes in the gastrointestinal tract due to intrinsic adaptations of performing PA [51]. Interestingly, from an ecological perspective, microbial diversity may be a key factor in allowing an ecosystem to continue operating properly [52]. In fact, greater species diversity has been associated with a healthy phenotype’s host [53]. This is due to the potential effects that the bacteria can exert via metabolites, such as short-chain fatty acids and neurotransmitters locally and extra-intestinal tissues in the host [54].

Our data showed that the participants with low time spent in vigorous PA had higher relative abundance of the *Gammaproteobacteria* class than individuals with higher time spent in vigorous PA. The relative abundance of the *Gammaproteobacteria* class (*Proteobacteria* phylum) has been reported to be increased in obese mice [55] and individuals with obesity [56], and disease states such as metabolic diseases and intestinal inflammation [57]. In fact, many common human pathogens, known as sulphur producers [58], are found in the *Gammaproteobacteria* class, for example, *Escherichia, Shigella*, and *Yersinia* genera [58]. In agreement with our findings, sedentary women and participants with low cardiorespiratory fitness [59] had a higher relative abundance of the *Gammaproteobacteria* class than active women and participants with high cardiorespiratory fitness, respectively. Similarly, a very recent study performed in > 8,000 individuals using accelerometers observed that PA levels were associated differently with faecal microbiota composition, suggesting that the higher the PA level is, the higher is the diversity [60]. Moreover, several studies have shown that exercise seems to decrease the relative abundance of the *Gammaproteobacteria* class [61, 62]. Thus, our results suggest that performing less than 1 min/day of vigorous PA could be related to having a higher relative abundance of the *Gammaproteobacteria* class, bacteria considered health-detrimental.

In contrast, we observed that individuals with high and intermediate time spent in vigorous PA had a higher relative abundance of the *Porphyromonadaceae* family and *Alistipes* genus (both *Bacteroidetes* phylum) than individuals with lower time spent in vigorous PA. Accordingly, in a cross-sectional study of professional martial arts athletes, the relative abundance of the *Porphyromonadaceae* family was higher in the higher-level athletes in comparison with the lowerlevel athletes [63]. Moreover, regular swimming training [64] and voluntary wheel running [65], both in mice, were able to increase the relative abundance of the *Porphyromonadaceae* family. In fact, it has recently been found that lean individuals had a significantly higher relative abundance of the *Porphyromonadaceae* and *Rikenellaceae* families than individuals with obesity [45]. Of note is the fact that the *Alistipes* genus belongs to the *Rikenellaceae* family. In resistance-trained mice, the relative abundance of the *Alistipes* genus was positively correlated with resistance performance [66]. In humans, the relative abundance of the *Alistipes* genus is increased after consuming an animal-based diet intake, rich in protein, for 5 days [64]. Certain species that belong to the *Alistipes* genus are involved in amino acid metabolism; specifically, they can hydrolyse tryptophan to indole [67]. Since tryptophan is an essential amino acid that cannot be produced by animal cells, humans rely on dietary intake, mainly proteins, for incorporating it into the organism [68]. In our study, the individuals with intermediate time spent in vigorous PA had higher protein intake than individuals with low time spent in vigorous PA. In fact, when the protein intake was included as a confounder, the differences in the relative abundance of the *Alistipes* genus between these individuals disappeared. Considering the relationship between the *Alistipes* genus and protein metabolism [67], and the results observed in the present study, it seems possible that these differences were explained by protein intake. Therefore, our data suggest that spending time on vigorous PA, in the range 3–14 min/day, could be related to having a higher relative abundance of *Porphyromonadaceae* family bacteria, whereas the protein intake seems to modulate the relative abundance of the *Alistipes* genus in individuals with intermediate time spent in vigorous PA. Even so, the possible effect of time spent in vigorous PA on the relative abundance of the *Gammaproteo-bacteria* class, *Porphyromonadaceae* family and *Alistipes* genus deserves further analysis.

### Limitations and strengths

A limitation to consider in the current study is that it followed a cross-sectional design, which prevents a causal interpretation of our results. Well-designed randomized controlled trials should be carried out to elucidate the role of PA in faecal microbiota diversity and composition. In addition, we do not know whether our findings apply to older people or individuals presenting any metabolic disease. As for strengths of this study, we sequenced the microbiota composition using the latest technology (Illumina platform) and annotations were made with RDP to the genus taxon level. Moreover, PA was objectively measured by accelerometry during 7 consecutive days (24 h/day) [21], and we used a cut-point-free approach to assess overall PA since PA intensities estimated from cut-points might be biased by poor calibration studies [69].

## CONCLUSIONS

Our data showed that overall PA and time spent in vigorous PA were positively correlated with faecal microbiota diversity in young adults. Moreover, the individuals with low time spent in vigorous PA presented higher relative abundance of the *Gammaproteobacteria* class, whereas the individuals with high time spent in vigorous PA had higher relative abundance of the *Porphyromonadaceae* family. Altogether, these findings suggest that PA, especially of vigorous intensity, is related to faecal microbiota diversity and the *Gammaproteo-bacteria* class and *Porphyromonadaceae* family in young adults. Further studies are needed to confirm this relationship.

## Data Availability

The datasets generated during the current study are available from the corresponding author on reasonable request.

## Clinical Trial Information

ClinicalTrials.gov no. NCT02365129 (registered 18 February 2015).
